# Temporal Proteome and Lipidome Profiles Reveal Hepatitis C Virus-Associated Reprogramming of Hepatocellular Metabolism and Bioenergetics

**DOI:** 10.1371/journal.ppat.1000719

**Published:** 2010-01-08

**Authors:** Deborah L. Diamond, Andrew J. Syder, Jon M. Jacobs, Christina M. Sorensen, Kathie-Anne Walters, Sean C. Proll, Jason E. McDermott, Marina A. Gritsenko, Qibin Zhang, Rui Zhao, Thomas O. Metz, David G. Camp, Katrina M. Waters, Richard D. Smith, Charles M. Rice, Michael G. Katze

**Affiliations:** 1 Department of Microbiology, School of Medicine, University of Washington, Seattle, Washington, United States of America; 2 Laboratory of Virology & Infectious Disease, Center for the Study of Hepatitis C, Rockefeller University, New York, New York, United States of America; 3 Environmental Molecular Sciences Laboratory, Pacific Northwest National Laboratory, Richland, Washington, United States of America; 4 Computational Biology & Bioinformatics, Pacific Northwest National Laboratory, Richland, Washington, United States of America; 5 Washington National Primate Research Center, University of Washington Seattle, Washington, United States of America; University of Kentucky College of Medicine, United States of America

## Abstract

Proteomic and lipidomic profiling was performed over a time course of acute hepatitis C virus (HCV) infection in cultured Huh-7.5 cells to gain new insights into the intracellular processes influenced by this virus. Our proteomic data suggest that HCV induces early perturbations in glycolysis, the pentose phosphate pathway, and the citric acid cycle, which favor host biosynthetic activities supporting viral replication and propagation. This is followed by a compensatory shift in metabolism aimed at maintaining energy homeostasis and cell viability during elevated viral replication and increasing cellular stress. Complementary lipidomic analyses identified numerous temporal perturbations in select lipid species (e.g. phospholipids and sphingomyelins) predicted to play important roles in viral replication and downstream assembly and secretion events. The elevation of lipotoxic ceramide species suggests a potential link between HCV-associated biochemical alterations and the direct cytopathic effect observed in this *in vitro* system. Using innovative computational modeling approaches, we further identified mitochondrial fatty acid oxidation enzymes, which are comparably regulated during *in vitro* infection and in patients with histological evidence of fibrosis, as possible targets through which HCV regulates temporal alterations in cellular metabolic homeostasis.

## Author Summary

As parasites, viruses rely on the cells they infect to provide the energy and building blocks required for their survival and propagation. However, relatively little is known about the extent to which viruses modulate host cell metabolism and the consequences of these disruptions. Here we integrate proteomic and lipidomic profiling with computational modeling approaches to probe the impact of HCV infection on the global metabolism of cultured hepatoma cells, and to understand the potential contribution of such perturbations to viral pathogenesis. Our findings suggest that increases in host catabolic and biosynthetic activities occur early during infection, providing the host macromolecules necessary for viral growth. As infection progresses and cellular stress increases, however, a compensatory metabolic shift occurs in an attempt to maintain energy homeostasis and cell viability. This shift is accompanied by changes in lipid abundance, which has predicted consequences for the viral life cycle and pathogenesis. Computational modeling was then used to identify novel host proteins functioning as key regulators of this HCV-associated metabolic reprogramming. In summary, these studies provide new insights into the marked disruption of cellular metabolic homeostasis that occurs during HCV infection and that may contribute to liver disease progression.

## Introduction

Persistent infection with hepatitis C virus (HCV), a single-stranded positive RNA virus of the *Flaviviridae* family, is a major cause of liver disease and a global public health problem. Chronically infected individuals develop variable degrees of hepatic inflammation and fibrosis, and are at increased risk for developing cirrhosis and hepatocellular carcinoma [1]. Current therapy consists of a combination of drugs that target cellular functions, including pegylated-interferon to boost the interferon-mediated antiviral response, and ribavirin, a nucleoside analog that suppresses HCV replication by impairing guanine nucleotide biosynthesis [2]. Unfortunately, this treatment regimen has limited efficacy, especially for certain HCV genotypes and patient populations, and its poor tolerability often leads to discontinuation.

All viruses rely on constituents of the host cell to provide the energy, macromolecules and structural organization necessary for their propagation. This dependence on host interactions has led to significant interest in better understanding those pathways/processes crucial to the viral life cycle, as these represent potential targets for new antiviral strategies [2]–[4]. HCV infection has long been associated with abnormalities in lipid metabolism, and lipids have been shown to play important roles in various aspects of the virus life cycle [2],[3],[5]. For example, the biosynthesis of cholesterol, fatty acids, and geranylgeranyl and sphingolipid species is key to HCV replication, presumably by promoting the formation of lipid rafts on which replicase complexes assemble [6]–[10]. The development of a cell culture system that supports not only HCV replication but also the production of infectious virus has revealed additional roles for lipid metabolism in viral particle assembly, secretion and infectivity. Lipid droplets have been shown to function in the assembly of infectious particles, and HCV production is further dependent on apolipoprotein B (apoB) expression and very low density lipoprotein (VLDL) assembly and secretion [11]–[13]. The association of HCV morphogenesis with VLDL production has led to the identification of new cellular targets (e.g. apoB, microsomal triglyceride transfer protein, and long chain acyl –coenzyme A synthetase 3) with the potential to limit both processes [12]–[15]. Lipidomic analyses of mature virions isolated from infected-cell culture supernatants suggest that the HCV membrane is enriched in cholesterol; modulation of the virion-associated cholesterol or sphingomyelin composition alters infectivity by inhibiting virus internalization [16]. Host cell lipid metabolism is therefore critical for multiple stages of the HCV life cycle, and represents an important area for the exploration of new antiviral reagents.

Despite the demonstrated importance of lipid components, the extent to which HCV modulates global intracellular metabolism to create an environment for RNA replication and production of progeny particles is currently unknown. Here we use liquid chromatography-mass spectrometry (LC-MS) together with the AMT tag approach to identify alterations in the host cell proteome and lipidome occurring in response to *in vitro* infection of Huh-7.5 cells with a chimeric HCV genotype 2a virus, J6/JFH-1. Our data reveal a temporal sequence of modifications to the host proteome that were not predicted from our previous gene expression analyses [17], suggesting that HCV dramatically disrupts cellular metabolic homeostasis via post-transcriptional regulatory mechanisms. We also observe global changes in lipid abundance, which are predicted to impact the HCV life cycle and pathogenesis. We further describe a computational modeling approach, which uses these high-throughput datasets to infer regulatory and functional relationship networks that provide information about the systems-level role of key proteins and lipids important for HCV-associated metabolic reprogramming.

## Materials and Methods

### Generation of Cell Culture Virus and Experimental Infections

Approximately 7×10^6^ Huh-7.5 cells (human hepatoma cell line) were electroporated with 2 µg of *in vitro* transcribed RNA, representing the chimeric HCV genome J6/JFH-1 [18]. One source of virus used for the infection experiments consisted of a pool of adapted virions obtained from cell culture supernatants that had been passaged multiple times following electroporation (HCVcc ‘pool’; refer to [17]). Alternatively, a J6/JFH-1 chimeric genome containing twelve cell culture adaptive mutations (HCVcc ‘clone’), isolated from the HCVcc ‘pool’, was used to generate virus stocks directly from electroporated cell supernatants by collecting samples every 12 h over 2–5 days post-electroporation. Both sources of virus demonstrated enhanced viral kinetics (>10-fold viral titer compared to parent; manuscript in preparation, Rice CM) that was necessary to infect the large number of cells required for the proteomic analysis. Parallel electroporations were performed in the absence of HCV RNA to generate a control supernatant sample (‘mock’). In addition, the HCV stock was used to generate a UV-inactivated, non-infectious control (UV-HCVcc; refer to [17]). For infection experiments (n = 4), low passage Huh-7.5 cells were seeded at a density of ∼3×10^6^ cells/p150 plate, to ensure that cells would not reach confluency by the time of harvest, and treated for ∼8–12 h with 20 ml of supernatant containing virus (HCVcc), UV-inactivated virus (UV-HCVcc), or conditioned media (mock, CM). Following initial exposure, the supernatant was replaced with fresh media and incubated until harvest at 24, 48, or 72 h post-infection. The multiplicity of infection (MOI) for the experiments was ∼1–2 and resulted in >50% of cells being infected by 24 hours after HCVcc exposure.

### Harvesting Cells After Virus Infection

Following removal of supernatant, cells were washed once with PBS and then scraped from the plate(s) in ice cold PBS. Identically treated cells were pooled from replicate plates, if necessary, to obtain approximately 10^7^ total cells per time point per condition. The cells were subsequently split into several fractions: 10% for RNA isolation for previously reported genomics analyses [17], 10% for lipidomic analysis, and 80% for peptide generation/proteomic analysis.

### Lipidomic Sample Preparation

Cells were pelleted in a siliconized microfuge tube, followed by disruption with chloroform∶methanol (2∶1 ratio), and pre-chilled to −20°C. After several rounds of vortexing on ice, samples were centrifuged at 2,000 RPM for 10 minutes to separate a water-soluble fraction (upper layer) and a lipid soluble fraction (lower layer). Both fractions were dried in a speed vac and stored at −80°C until analysis.

### Proteomic Sample Preparation

Cells were washed in 0.5 x PBS, pelleted, and stored at −80°C until sample preparation. Samples from all time points were prepared on the same day, initially by lysing the thawed cell pellets in hypotonic buffer (5 mM K_3_PO_4_) at room temperature. Samples were solubilized and denatured by adding trifluoroethanol (TFE) to achieve a final concentration of 50%, followed by sonication on ice and incubation at 60°C for 1 h along with additional sonication. An equal volume of 500 mM NH_4_HCO_3_ (pH 8.0) was added to adjust to pH 8.0, and 5 mM tributylphosphine (TBP) was then added for 1 h at 37°C to allow reduction of disulfide bonds. Following centrifugation, soluble material was transferred to a fresh tube and the volume of sample was reduced to ∼100 µl using a speed vac. Additional NH_4_HCO_3_ (50 mM) was then added to reduce the amount of TFE in the samples to <10%. Protein concentration was determined using BCA protein assay (Thermo Scientific) and the denatured and reduced proteins were subsequently digested in sequencing-grade trypsin (50∶1–100∶1, w/w; Promega #V5111) for ∼6 h at 37°C. Peptide concentration was then determined using BCA protein assay and samples were stored at −80°C until analysis. Prior to mass spectrometric analysis, all peptide samples were loaded on a 1 ml solid-phase extraction (SPE) C_18_ column (Supelco, Bellefonte, PA) and washed with 4 ml of 0.1% trifluoroacetic acid (TFA)/5% acetonitrile (ACN). Peptides were eluted from the SPE column with 1 ml of 0.1% TFA/80% ACN and lyophilized.

### Trypsin-Catalyzed ^16^O/^18^O Labeling of Huh-7.5 Cell Culture Protein Digests

Prior to trypsin-catalyzed ^16^O/^18^O labeling the samples were reconstituted in 25 mM ammonium bicarbonate (NH_4_HCO_3_) and the peptide concentration was measured using the BCA protein assay (Pierce) in order to normalize for protein content. Trypsin-catalyzed ^16^O/^18^O labeling was carried out as described previously [19],[20]. Peptides from time-matched mocks (CM) were individually labeled with ^18^O, and spiked at equal amounts into the appropriate HCVcc- or UV-HCVcc-inoculated sample for analysis of protein abundance changes occurring at 24, 48 and 72 h post-infection. In each case a total of 25 µg of the resulting peptide mixture was subsequently used for pre-MS peptide separation and quantitative LC-MS peptide analysis as described in the following sections.

### Pre-MS Peptide Separation

Labeled and combined Huh-7.5 peptide samples, total of 25 µg peptide for each sample, were subjected to strong cation exchange chromatography (SCX) prior to LC-MS analysis as previously described [21]. Briefly, samples were suspended in 1.5 ml of 10 mM ammonium formate, 25% acetonitrile, pH 3.0 and injected onto a 10×4.6-mm guard column attached to a polysulfoethyl A 200×4.6-mm (5-µm, 300-Å) column (Poly LC, Columbia, MD). The mobile phases consisted of solvent A (10 mM ammonium formate, 25% acetonitrile, pH 3.0) and solvent B (500 mM ammonium formate, 25% acetonitrile, pH 6.8). The separations were performed using an Agilent 1100 series HPLC system at a flow-rate of 200 µL/min. After sample loading, the separation was isocratic for 5 min with 100% solvent A with a flow rate of 1 ml/min. Peptides were eluted using sequential linear gradients from 100% solvent A to 50% solvent B over 25 min and from 50% solvent B to 100% solvent B over another 10 min. The mobile phase was held at 100% solvent B for another 10 min. A limited number of 5 fractions were collected and retained for each separation based upon the observed reproducible chromatographic peaks, lyophilized and analyzed via reversed-phase LC-MS.

### Preparation of Packed Capillary Columns for LC-MS Peptide and Lipid Analyses

Seventy-five or 150 µm capillary LC columns were slurry-packed with either 3 or 5 µm Jupiter C18-RP particles (Phenomenex, Torrance, CA) for peptide and lipid analyses, respectively, as previously described [22]. Briefly, the stationary phase was added to a stainless steel reservoir, to which an empty fused silica capillary (Polymicro Technologies, Phoenix, AZ) was connected. The opposite end of the capillary was connected to a stainless steel union (Valco, Houston, TX) containing a stainless steel screen (either 0.5 or 2 µm mesh for 75 or 150 µm columns, respectively, Valco) that served as a frit. Acetonitrile was used as the packing solvent and was delivered at constant pressure by syringe pump (ISCO, Lincoln, NE). Initially, a pressure of 100 psi was applied. The pressure was then increased stepwise to and held constant at 10,000 psi or 7,000 psi for 75 or 150 µm columns, respectively, for 5–10 min under sonication.

### LC-MS Peptide Analysis

Each Huh-7.5 SCX fractionated peptide sample was individually analyzed using a fully automated custom built capillary LC system containing four column 75 µm×65 cm capillary column system, as previously described [23]. The capillary LC system was coupled to a hybrid linear ion-trap-orbitrap (LTQ-Oribitrap, ThermoFisher, San Jose, CA) and analysis was performed as previously described [19],[20].

### LC-MS Lipid Analyses

An automated LC system with two 150 µm×65 cm capillary columns was used. Dried lipid extracts were reconstituted in 75 µl methanol and vortexed for 10 s. The samples were then centrifuged at 13,400×g for 5 min to remove any particulates. Lipid molecular species were chromatographically separated as previously described [24].

The capillary LC system was coupled to a hybrid linear ion-trap-Fourier transform ion cyclotron resonance (FTICR) mass spectrometer (LTQ-FT, ThermoFisher, San Jose, CA). The capillary temperature and electrospray voltage were 200°C and +2.2 kV, respectively. The FT was used as the mass analyzer during MS survey scans over the *m/z* range 300–2000, with a duty cycle of ∼1.0 s. Data-dependent MS/MS was performed in the LTQ for the top 5 ions, with a normalized collision energy of 35%. Dynamic exclusion in the LTQ during data-dependent MS/MS experiments was enabled as follows: repeat count of 2, repeat duration of 30 s, exclusion list size of 250, and exclusion duration of 60 s.

### Processing of Peptide and Lipid LC-MS Datasets

Both peptide and lipid LC-MS datasets, defined as the data obtained from a single LC-MS analysis, were processed using the PRISM Data Analysis system [25], a series of software tools (e.g. Decon2LS [26], VIPER [27]; freely available at http://ncrr.pnl.gov/software/) developed in-house. The first step involved deisotoping of the raw MS data to give the monoisotopic mass, charge state, and intensity of the major peaks in each mass spectrum. The data were next examined in a 2-D fashion to identify groups of mass spectral peaks that were observed in sequential spectra using an algorithm that computes a Euclidean distance in n-dimensional space for combinations of peaks. Each group, generally ascribed to one detected species and referred to as a “feature”, has a median monoisotopic mass, central normalized elution time (NET), and abundance estimate computed by summing the intensities of the MS peaks that comprise the entire LC-MS feature.

The identities of detected features of both peptides and lipid LC-MS datasets were initially determined by comparing their measured monoisotopic masses and NETs to the calculated monoisotopic masses and observed NETs of each of the peptides or lipids in an accurate mass and time (AMT) tag database within search tolerances of ±5 ppm and ±0.02 NET for monoisotopic mass and elution time, respectively [24]. The AMT tag database utilized for peptide matching was a composite of all previous published Huh-7.5 and human liver tissue MS/MS analyses [20],[21]. In contrast, the lipid AMT tag database was constructed from human plasma, erythrocyte, and lymphocyte lipids. Non-linear chromatographic alignment of LC-MS datasets was performed with the LCMSWARP algorithm [28] during database matching by using the NETs of either peptide or lipid AMT tags as retention time locks. The identities of some features that did not match entries in the lipid AMT tag database were determined manually based on accurate mass, isotopic distribution (using the in-house software IsotopicDistributionModeler), and MS/MS information, as previously described [24].

In regard to peptide data analysis, the abundance ratios (^18^O/^16^O) for labeled peptide pairs were accurately computed using an equation as previously reported [19],[29]. All ratios corresponding to peptide sequences which overlapped between multiple protein groups, based upon ProteinProphet results [30], were removed as the exact protein source of these peptide sequences is ambiguous. After rolling-up all remaining quantified peptides into non-redundant protein groups using the ProteinProphet results, the corresponding ^18^O/^16^O intensity data was loaded into Rosetta Elucidator (Rosetta Biosoftware, Seattle, WA) and an error-model for ^18^O-labeled FTICR data was applied as previously described [20]. Ratios from multiple observations of the same protein across the 5 SCX fractions were then rolled up to compute a final protein abundance ratio for all proteins identified in a given sample and to identify those proteins exhibiting statistically significant (p≤0.05) changes in abundance compared to the control sample.

For lipid analysis, after chromatographic alignment and database matching, intensity normalization was applied using the expectation maximization algorithm [24]. Briefly, this algorithm analyzes the histogram of log ratios of intensities of features common to two or more datasets and finds the peak apex of this distribution by assuming that the histogram is a mixture of a normal density corresponding to unchanged features and uniform density background corresponding to changed features. The expectation maximization algorithm calculates the normal and uniform parts of the histogram, and a shift in intensity is applied to all features in the aligned dataset. It is important to note that all lipid features (*i.e.* both identified and unidentified) were considered during intensity normalization.

The set of normalized lipid features (both identified and unidentified) was then transformed to log 2 scale and comparative data analysis was performed on two levels. The first level considered only complete data, i.e. those lipid features detected in every LC-MS dataset. The second level allowed for some missing data; a feature was required to be observed in both LC-MS replicates of two out of three conditions (mock, HCVcc, and UV-HCVcc). It is important to note that more observations than the required minimum were present for most lipid features within a culture condition. The data matrices corresponding to these two levels were analyzed separately using Matlab, and changes in the lipid profiles as functions of time and condition were determined using analysis of variance (ANOVA, p<0.05). Lipid features that were significantly different by ANOVA were further analyzed using principal component analysis (PCA) [31]. Abundance values for missing lipid features were estimated as the average lipid abundance obtained from the same features observed in the remaining LC-MS datasets to aid visualization in PCA only. Finally, a table showing the associated p values, average lipid abundances, lipid abundance standard deviations, mass-to-charge (m/z) ratio, NET, and lipid identities were generated and are provided as supplementary data.

### Network Generation and Topological Analysis

We generated correlation networks by calculating Pearson correlation coefficient between the abundance profiles of all pairs of proteins and/or lipid species. We filtered out relationships between proteins in which more than two time points did not contain an abundance measurement in either profile. Human protein-protein interactions were obtained from http://cytoscape.wodaklab.org/wiki/Data_Sets and were filtered to include only interactions between proteins observed by proteomics. Networks were merged by filtering at various thresholds and using the union of edges in the ‘parent’ networks to generate the ‘child’ network. Network topology was calculated using the NetworkX Python module (http://networkx.lanl.gov/) using betweeness centrality. Bottlenecks were considered to be the top 20% of proteins/lipids as ranked by betweeness (as in [32]–[34]), though all observations were similar using 10% and 5% thresholds. Statistical significance was calculated using a chi-square test and p values less than 0.05 were considered significant.

Both the International Protein Index database accession number (IPI identifier; commonly used for proteomics) and the corresponding EntrezGene identifier are listed here for each protein mentioned in the text. LRPAP1- IPI00026848, EntrezGene 4043; PEBP1- IPI00219446, EntrezGene 5037; APOE- IPI00021842, EntrezGene 348; PAFAH1B3- IPI00014808, EntrezGene 5050; ARPC3- IPI00005162, EntrezGene 10094; CHP- IPI00218136, EntrezGene 11261; VAPA- IPI00170692, EntrezGene 9218; IK- IPI00011875, EntrezGene 3550; PSMA5- IPI00291922, EntrezGene 5686; PSME1- IPI00030154, EntrezGene 5720; PSME3- IPI00030243, EntrezGene 10197; USP39- IPI00419844, EntrezGene 10713; TPI1- IPI00465028, EntrezGene 7167; LDHA- IPI00217966, EntrezGene 3939; PKLR- IPI00027165, EntrezGene 5313; TKT- IPI00021716, EntrezGene 7086; TALDO1- IPI00024102, EntrezGene 6888; UMP-CMPK- IPI00219953, EntrezGene 129607; ITPA- IPI00018783, EntrezGene 3704; TS- IPI00103732, EntrezGene 7298; POLD1- IPI00002894, EntrezGene 5424; AK4- IPI00016568, EntrezGene 205; CS- IPI00025366, EntrezGene 1434; IDI1- IPI00220014, EntrezGene 3422; FABP1- IPI00010292, EntrezGene 2168; TMEM97- IPI00020004, EntrezGene 27346; FASN- IPI00418433, EntrezGene 2194; ACOX1- IPI00296907, EntrezGene 51; DECR1- IPI00003482, EntrezGene 1666; HADH2- IPI00017726, EntrezGene 3028; DCI- IPI00300567, EntrezGene 1632; ECH1- IPI00550041, EntrezGene 1891; OAT- IPI00022334, EntrezGene 4942; GLUD1- IPI00016801, EntrezGene 2746; FH- IPI00296053, EntrezGene 2271; MDH2- IPI00291006, EntrezGene 4191: GOT2- IPI00018206, EntrezGene 2806; IDH2- IPI00011107, EntrezGene 3418; SOD1- IPI00218733, 6647; PRDX1- IPI00000874, EntrezGene 5052; TXN- IPI00216298, EntrezGene 7295.

## Results

### Global Proteome Response to Acute HCV Infection of Cultured Hepatoma Cells

**Figure 1:**
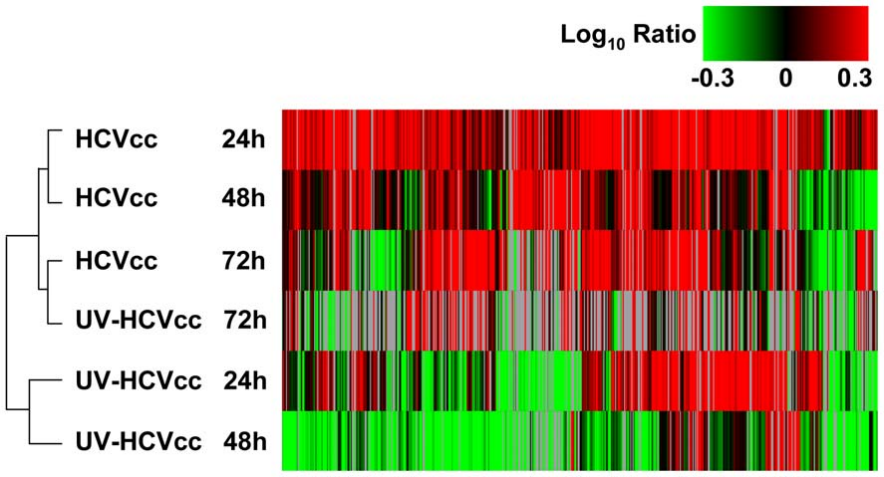
HCV infection induces temporal changes in the host cell proteome. Data from the five strong cation exchange (SCX) fractions generated and analyzed for each sample were processed and loaded into Rosetta Elucidator (Rosetta Biosoftware Inc.) for error modeling and computation of final protein abundance ratios. Among 2,418 unique proteins quantified, a total of 495 proteins differentially regulated (≥1.5-fold change, p-value≤0.05) in at least two experiments were selected for subsequent cluster analysis. Two-dimensional hierarchical clustering was performed with a weighted average method and Cosine correlation metric. Each *horizontal row* represents an individual infection condition and each *vertical column* an individual protein (495 total). Abundance ratios were monitored 24, 48 and 72 h after infection with either HCVcc (chimeric HCV 2a virus, J6/JFH-1) or UV-HCVcc (UV-inactivated chimeric HCV 2a virus, J6/JFH-1) and using a time-matched mock (conditioned media, CM) as the reference. Protein abundance ratios are colored according to the fold changes and the *color scale* indicates the magnitude of fold change. Black squares indicate no change in protein abundance. Gray squares indicate missing data.

In order to gain a better understanding of virus-host interactions during the early phase of acute infection, quantitative proteomic studies were carried out in Huh-7.5 cells infected with an HCV cell culture (HCVcc) genotype 2a chimeric virus, J6/JFH-1. Changes in host protein abundance in HCVcc-infected cells were compared to cells inoculated with conditioned media (CM) or exposed to UV-inactivated HCVcc, which cannot undergo replication. Using^ 16^O/^18^O stable isotope labeling together with the AMT tag approach [20],[35], we quantified a total of 2,418 proteins (Supplementary Table S1). Of these, 495 proteins exhibiting significant abundance changes (≥1.5-fold, p≤0.05) between 24–72 h post-infection were analyzed in Spotfire (Spotfire, Inc.) using a hierarchical clustering algorithm that allowed grouping of similar samples based on the pattern of fold-change in protein abundance (Fig. 1, Supplementary Table S1).

Interestingly, we found that cells inoculated with UV-inactivated virus underwent dramatic changes in their proteome that partially overlapped with the altered protein abundances detected in HCVcc-infected cells (Fig. 1). These findings suggest that HCV binding to the hepatocyte plasma membrane induces a host cell response. Examination of this subset of proteins revealed conserved increases across a broad range of cellular processes, indicative of both pro-viral (e.g. lipid/lipoprotein binding and hydrolysis- LRPAP1, PEBP1, APOE, PAFAH1B3; clathrin-mediated endocytosis- ARPC3, CHP; tight junction signaling- VAPA; antagonism of host defenses- IK) and anti-viral (e.g. ubiquitination and MHC class 1 antigen presentation- PSMA5, PSME1, PSME3, USP39) activities (Supplementary Table S1). Despite these similarities, the clustering analysis segregated HCV-cc and UV-inactivated HCVcc-inoculated cells into two distinct groups, delineating subsets of proteins that increased in abundance specifically in response to productive HCV infection (Fig. 1). Since we were particularly interested in understanding the host processes that control HCV replication and infectious particle production, we further explored the biological significance of the HCVcc-specific subsets using Ingenuity Pathways Analysis (IPA) (Ingenuity Systems Inc). The results of this functional and canonical pathways analysis are described in the sections that follow.

### HCV Infection Induces an Early Metabolic Re-Routing that Favors Elevated Host Biosynthetic Activities Important for the Viral Life Cycle

**Figure 2:**
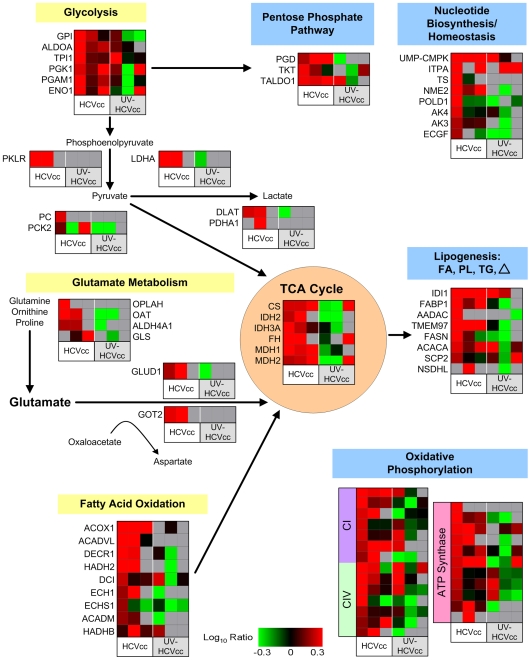
HCV induces perturbations in metabolic homeostasis. The 495 differentially regulated proteins described in Figure 1 were subjected to functional and canonical pathway analysis using Ingenuity Pathways Analysis (Ingenuity Systems Inc). Shown here is a metabolic map illustrating the dynamic changes in host biosynthetic and catabolic pathways occurring during HCV infection. Each *horizontal row* represents a differentially regulated protein identified by its gene symbol and each *vertical column* represents an individual infection condition. Abundance ratios for the various samples appear in the following order from left to right: infection with HCVcc at 24, 48 and 72 h (columns 1, 2, and 3, respectively) or UV-HCVcc at 24, 48 and 72 h (columns 4, 5, and 6, respectively). Protein abundance ratios are colored according to the fold changes and the *color scale* indicates the magnitude of fold change. Black squares indicate no change in protein abundance. Gray squares indicate missing data. HCV infection induces a unique proteomic profile indicative of marked disruptions in cellular metabolic homeostasis. The limited overlap, and in some cases magnitude, of protein up-regulation in actively growing cells exposed to UV-inactivated virus further suggests that infectious HCVcc specifically institutes its own metabolic program by considerably stimulating host biosynthetic activities supporting viral growth. See text and Supplementary Table S2 for more detail including correlation of gene symbol with protein name.

Overall, the pathway analyses revealed a temporal sequence of modifications to the host cell proteome reflecting marked alterations in metabolic homeostasis during virus infection. The metabolic heat map presented in Figure 2 is a graphic display of the magnitude of the related protein abundance changes observed in either HCVcc or UV-inactivated HCVcc-inoculated cells, demonstrating the limited overlap in commonly regulated abundance differences. For example, of the 79 proteins represented here 59 were shown to be statistically significantly up-regulated (≥1.5-fold, p≤0.05) by infectious HCVcc at 24 h post-infection (highlighted in red in Supplementary Table S2). This contrasts with the smaller repertoire of differentially regulated proteins (17 total) observed in the presence of UV-inactivated HCVcc, which included 11 proteins exhibiting significant decreases in abundance and only 6 proteins exhibiting significant increases analogous to those seen with infectious HCVcc (highlighted in green and red in Supplementary Table S2, respectively). Notably, we detected a significant increase in the relative abundance of 6 out of 7 glycolytic enzymes in the presence of infectious HCVcc while only one of these proteins, triose phosphate isomerase 1 (TPI1), was also observed to be up-regulated in the presence of UV-inactivated virus (Fig. 2, highlighted in Supplementary Table S2). Our results indicate that the HCV-infected cell exhibits a unique proteomic (and metabolic) profile, considerably stimulating increased flux through the glycolytic pathway.

Analogous to the Warburg effect described for cancer cells and rapidly proliferating non-transformed cells [36],[37], we further observed proteome changes consistent with a shift toward lactate production rather than catabolism of glucose via the tricarboxylic acid (TCA) cycle. The concomitant increase in lactate dehydrogenase (LDHA; Fig. 2, Supplementary Table S2), is predicted to divert the majority of glycolytic pyruvate to lactate production and regeneration of NAD^+^ in support of continued glycolysis. Furthermore, the absence of a significant increase in pyruvate kinase (PKLR) abundance is expected to favor partitioning of glucose intermediates to host biosynthetic pathways supporting the viral life cycle. For example, accumulation of fructose 6-phosphate and glyceraldehyde 3-phosphate is predicted to promote rerouting into the pentose phosphate pathway for generation of ribose 5-phosphate, an important precursor for the synthesis of nucleotides required for RNA replication. Indeed, high levels of intracellular uridine triphosphate (UTP) and cytidine triphosphate (CTP) have been reported to be critical for HCV replication in non-dividing cells [38]. Consistent with this, we detected an increase in the relative abundance of non-oxidative pentose phosphate pathway enzymes, transketolase (TKT) and transaldolase (TALDO1), as well as several other proteins functioning in nucleotide synthesis and homeostasis [e.g. UMP-CMP kinase (UMP-CMPK), inosine triphosphate pyrophosphatase (ITPA), thymidylate synthase (TS), DNA polymerase delta catalytic subunit (POLD1), and adenylate kinase isoenzyme 4 (AK4)] (Fig. 2, Supplementary Table S2).

Although our results suggested HCV-infected cells exhibit a Warburg effect, we also observed a coordinated increase in the abundance of TCA cycle enzymes, as well as of numerous components of the electron transport chain and ATP-synthesizing proton pump, indicating that oxidative glucose metabolism pathways are intact (Fig. 2, Supplementary Table S2). Under conditions of sufficiently high glycolytic flux it is likely that some pyruvate is converted to acetyl-CoA and enters the TCA cycle even without a necessarily coordinate increase in PDH levels. The collective up-regulation of citrate synthase (CS) and various lipogenic proteins [e.g. isopentenyl-diphosphate delta isomerase 1 (IDI1), fatty acid binding protein 1 (FABP1), MAC30 protein (TMEM97), and fatty acid synthase (FASN)] 24 h after infection suggests that TCA cycle activity is coupled with the generation of cytosolic citrate for fatty acid synthesis, producing lipid species to support the viral life cycle (Fig. 2, Supplementary Table S2). Interestingly, we observed that the up-regulation of lipogenic enzymes was paralleled by increased abundance of proteins associated with peroxisomal and mitochondrial fatty acid oxidation [e.g. acyl-CoA oxidase 1 (ACOX1), 2,4-dienoyl-CoA reductase (DECR1), 3-hydroxyacyl-CoA dehydrogenase type II (HADH2), 3,2-trans-enoyl-CoA iosmerase (DCI),and ECH1 protein (ECH1)], suggesting active lipid synthesis and turnover (Fig. 2, Supplementary Table S2).

A final point worth addressing is the variation in magnitude of protein abundance changes observed here. It is reasonable to assume that the magnitude of abundance differences may provide an important perspective for biological interpretation and it is therefore not surprising that among the larger abundance increases we detected were those for nucleotide and lipid biosynthetic proteins of predicted importance to the viral life cycle (e.g. UMP-CMPK, ITPA, IDI1, and FABP1) (Fig. 2, Supplementary Table S2). However, we would like to emphasize that it is not clear to what extent protein abundance must be altered to exact a biologically relevant consequence. In this regard, the coordinate up-regulation, albeit to a lesser extent (∼1.5–3.0 fold change), of a majority of the enzymes in the glycolytic, non-oxidative pentose phosphate, and TCA pathways provides increased confidence in the significance of these protein abundance changes and their relative functional importance to HCV infection.

### Early Metabolic Re-Routing is Accompanied by Anaplerotic Reactions that Replenish Metabolic Intermediates and Sustain Macromolecule Biosynthesis

In order to ensure continued TCA cycle function in macromolecular biosynthesis, cells must replenish the pools of metabolic intermediates (anaplerosis) depleted during lipid, protein, and nucleic acid synthesis [36],[39]. For example, citrate export supporting lipid synthesis (e.g. fatty acid, cholesterol, and isoprenoids) results in a concomitant decline in oxaloacetate (OAA) from the TCA cycle, which must be replenished for further citrate production and lipogenesis to occur. Our proteomic data indicates that HCV-infected cells exhibit increased levels of several proteins involved in replenishing metabolic intermediates of the TCA cycle, including pyruvate carboxylase (PC), the major anaplerotic mitochondrial enzyme that generates OAA directly from pyruvate. In addition, we detected up-regulation of several enzymes catalyzing interconversion of ornithine, proline, and glutamate, suggesting that glutamine metabolism provides an alternative source of anaplerotic substrates (Fig. 2, Supplementary Table S2). This increased abundance of enzymes catalyzing glutamine flux through the latter half of the TCA cycle [e.g. ornithine aminotransferase (OAT), glutamate dehydrogenase (GLUD1), fumarate hydratase (FH), and mitochondrial malate dehydrogenase (MDH2)] provides substrates for ATP production while simultaneously replenishing OAA for biosynthesis. The further accumulation of aspartate aminotransferase (GOT2) suggests that a portion of the anaplerotic source of OAA is utilized for generating aspartate, a required precursor for nucleotide synthesis. Finally, the increased abundance of NADP-dependent isocitrate dehydrogenase (IDH2), an enzyme known to catalyze reductive carboxylation of glutamine and reverse TCA flux from α-ketoglutarate to citrate [40], suggests that glutamine metabolism may further contribute to anaplerosis by providing an alternative source of lipogenic acetyl-CoA.

### Acute HCV Infection is Associated with an Early Induction of Proteins Functioning in Cellular Stress Responses

**Figure 3:**
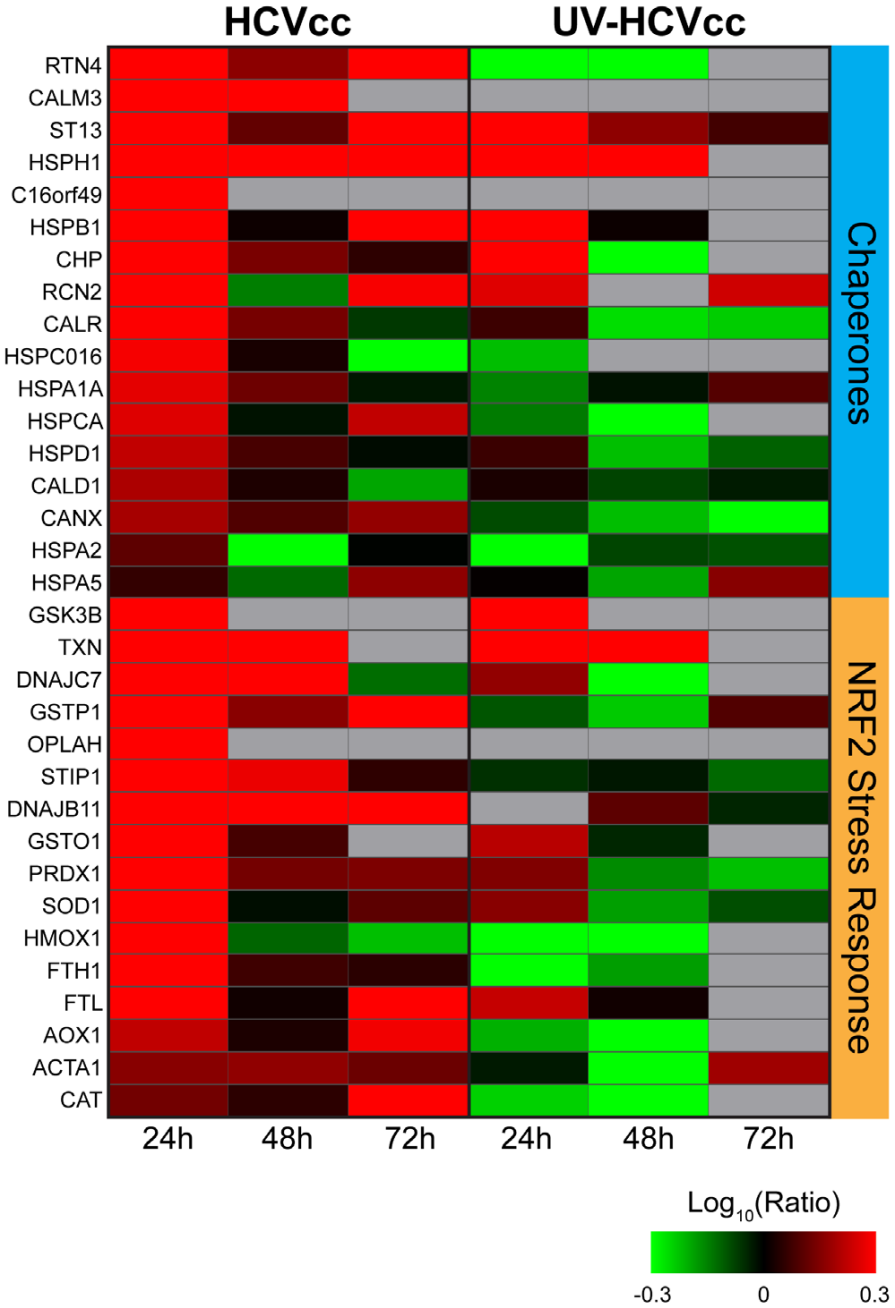
HCV induces protein abundance changes indicative of endoplasmic reticulum (ER) stress and oxidative stress during acute infection of cultured Huh-7.5 cells. Each *horizontal row* represents a differentially regulated protein identified by its gene symbol and each *vertical column* represents an individual HCV infection time point. Abundance ratios for the various samples appear in the following order from left to right: infection with HCVcc at 24, 48 and 72 h (columns 1, 2, and 3, respectively) or UV-HCVcc at 24, 48 and 72 h (columns 4, 5, and 6, respectively). Protein abundance ratios are colored according to the fold changes and the *color scale* indicates the magnitude of fold change. Black squares indicate no change in protein abundance. Gray squares indicate missing data. Refer to Supplementary Table S2 for more detail including correlation of gene symbol with protein name.

Consistent with previous reports [20],[41], we detected an early (24 h post-infection) induction of chaperone proteins and numerous components of the NF-E2-related factor-2 (Nrf2)-mediated oxidative stress response, providing evidence for endoplasmic reticulum (ER)/oxidative stress during acute infection of cultured Huh-7.5 cells (Fig. 3, Supplementary Table S2). Among the up-regulated antioxidant proteins were members of the reactive oxygen species (ROS) stress response [e.g. superoxide dismutase (SOD1) and peroxiredoxin 1 (PRDX1)] as well as proteins involved in maintaining the redox state of the cell [e.g. thioredoxin (TXN), various glutathione S transferases (GSTs)]. An accompanying increase in the abundance of numerous proteins functioning in translation initiation is in line with previous reports describing high levels of HCV and cellular protein synthesis despite the presence of ER stress (Supplementary Table S1) [41],[42].

### Remodeling of Metabolic Pathways Reflects a Shift from Synthetic to Energetic Purposes as Infection Progresses

Evaluation of later time points revealed that the increased abundance of fatty acid oxidation, amino acid catabolism, and TCA cycle enzymes was sustained 48 h after infection (Fig. 2, Supplementary Table S2). In stark contrast, the early up-regulation of proteins functioning in glycolysis, oxidative phosphorylation, and macromolecular biosynthesis was dramatically abated, suggesting a metabolic shift toward amino acid and fatty acid utilization for energy purposes through the TCA cycle. A similar metabolic phenomenon has been described for HepG2 cells, where rapidly proliferating (subconfluent) cells generate energy via glucose consumption and lactate production, along with *de novo* macromolecule synthesis, while growth arrested (confluent) cells exhibit increased amino acid flux through the TCA cycle [43]. Interestingly, our previously reported gene expression analyses, which were carried out in parallel with the current proteomic and lipidomic studies, demonstrated that HCV infection is associated with a progressive increase in the number of differentially regulated cell cycle checkpoint/arrest genes [17]. Subsequent flow cytometry analyses confirmed a decline in entry into S phase for infected cells [17]. Taken together, these findings suggest that HCV infection is associated with a delay in cell cycle progression that is accompanied by an adaptive metabolic response aimed at channeling substrates from synthetic to energetic purposes.

Review of the proteomic data at 72 h post-infection suggests a partial re-emergence of glycolytic and biosynthetic activities (Fig. 2, Supplementary Table S2). In this regard, we note that, despite our best efforts to obtain a high efficiency of infection, these cultures are characterized by asynchronous replication and we suspect that this re-emerging pattern reflects spread of infection.

### Alterations in Metabolic Homeostasis Occur During HCV Infection in Patients

**Figure 4:**
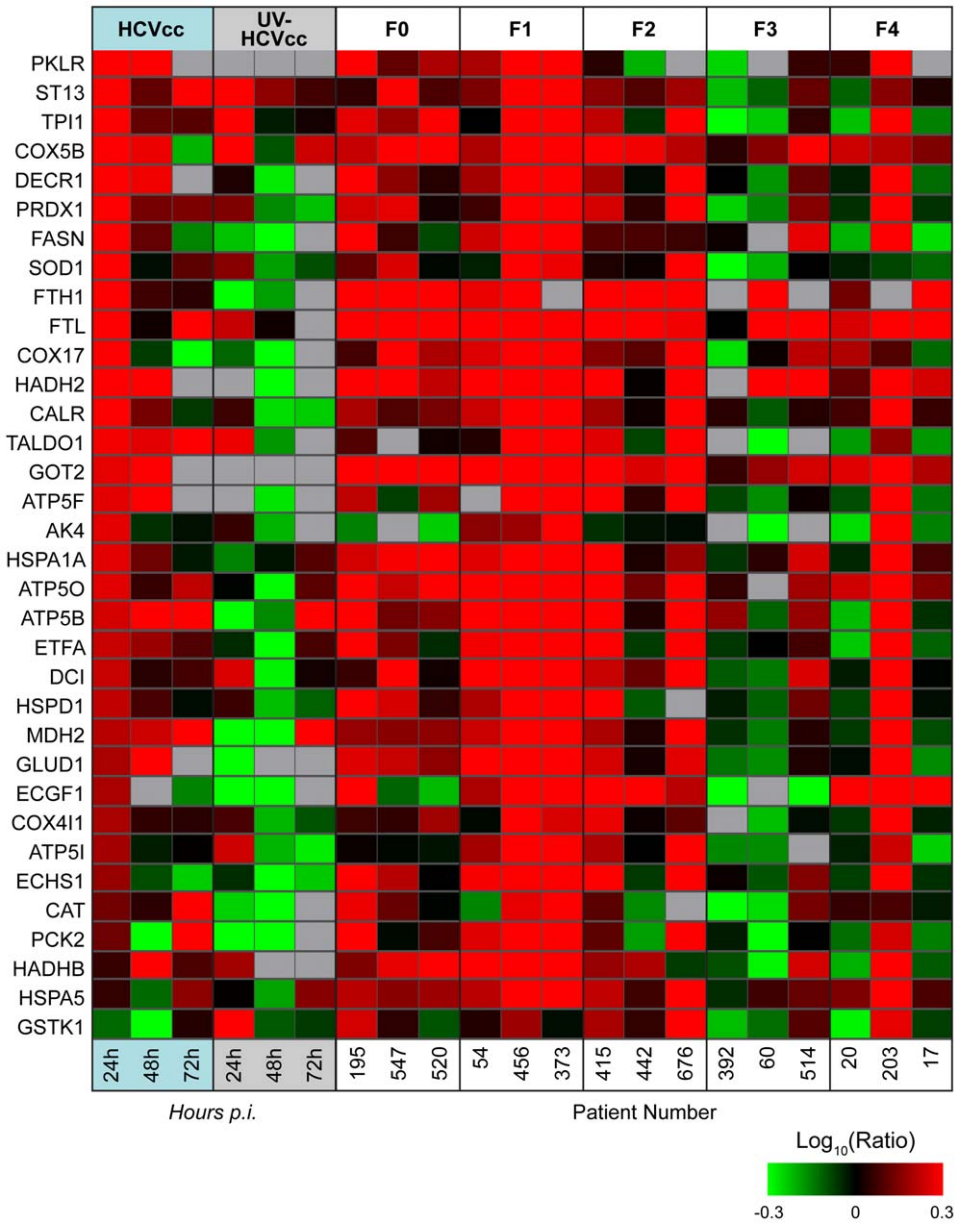
Common regulation of protein abundance changes in HCV-infected Huh-7.5 cells and patient liver tissue. Each *horizontal row* represents a differentially regulated protein identified by its gene symbol. Each *vertical column* represents an individual sample. Abundance ratios for the various samples appear in the following order from left to right: cultured hepatoma cells infected with HCVcc at 24, 48 and 72 h, cultured hepatoma cells exposed to UV-HCVcc at 24, 48 and 72 h, and then patients chronically infected with HCV and exhibiting stage 0 (F0), stage 1 (F1), stage 2 (F2), stage 3 (F3) or stage 4 (F4) fibrosis [20]. Protein abundance ratios are colored according to the fold changes and the *color scale* indicates the magnitude of fold change. Black squares indicate no change in protein abundance. Gray squares indicate missing data. Protein abundance information for HCVcc-infected Huh-7.5 cells can be found in Supplementary Table S2 and for human liver biopsy specimens in [20].

We have previously reported the global proteomic alterations accompanying liver disease progression in patients chronically infected with HCV [20]. To determine the clinical relevance of the protein abundance changes observed in HCVcc-infected Huh-7.5 cells, we compared the *in vitro* proteome data with the 210 clinically identified proteins exhibiting statistically significant (ANOVA) differences associated with fibrosis stage [20]. A subset of the carbohydrate, amino acid, and lipid metabolism proteins that were up-regulated early during HCV infection in cell culture (e.g. 24 h post-infection) were also up-regulated in chronically infected individuals with no or minimal liver disease (e.g. fibrosis stages F0-F2, Fig. 4). Further comparison revealed that the declines in fatty acid catabolism and oxidative stress responses evident at later times during HCV infection in cell culture (e.g. 72 h post-infection) coincide with similar decreases during liver disease progression *in vivo* (Fig. 4). These findings suggest that the proteome alterations in HCVcc-infected Huh-7.5 cells parallel those occurring during natural infection *in vivo*, reinforcing the utility of the *in vitro* system for investigating the mechanisms by which HCV modulates host cell metabolism and the interplay between altered cellular metabolic homeostasis and liver disease progression.

### Acute HCV Infection Induces Temporal Changes in the Host Cell Lipidome

**Figure 5:**
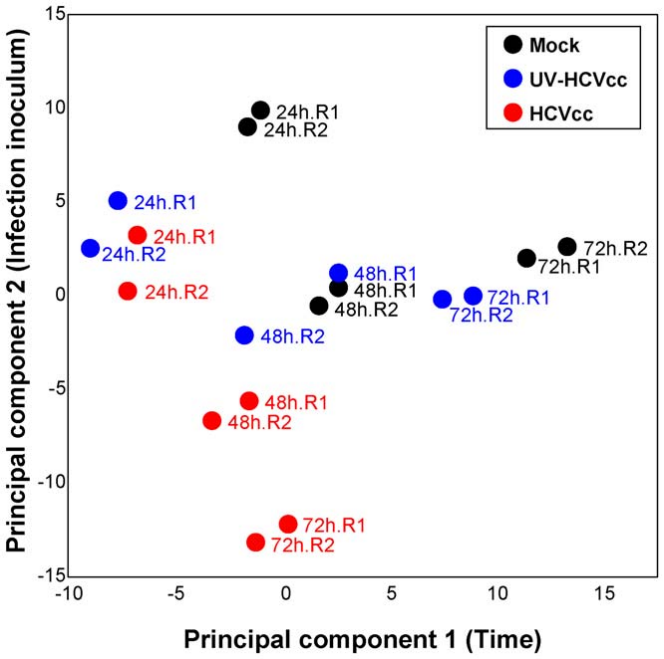
Principal components analysis scores plot showing the segregation of samples based on lipid profiles. Duplicate lipid abundance measurements (corresponding to R1 and R2) were made 24, 48 and 72 h after infection with either HCVcc or UV-HCVcc and using a time-matched mock (conditioned media, CM) as the reference. A total of 272 features significantly different (ANOVA, p<0.05) between treatment and time were compared using principal components analysis (PCA). The scores plot shows temporal differences (represented along principal component 1) as well as HCV-specific differences (represented along principal component 2).

The documented role of lipids in HCV infection, as well as the extensive changes in lipid metabolism predicted from our global proteome measurements, suggested significant impacts on the infected cell lipidome. We therefore conducted LC-MS-based studies to define the lipid profiles of HCVcc-infected Huh-7.5 cells, as well as cells exposed to CM or UV-inactivated virus, over time. Normalized abundances were averaged within respective replicates and statistical analysis was performed to identify 272 lipids with differential expression across conditions and time points (ANOVA, p<0.05; Supplementary Table S3) and, of these, 73 were further identified by matching to a lipid AMT tag database or by targeted MS/MS analysis (Supplementary Table S3). Subsequent comparison using principal components analysis (PCA) allowed the evaluation of trends or patterns across treatment conditions and time points (Fig. 5). Briefly, the greater the influence of time (principal component 1) or infection inoculum (principal component 2) on quantitative differences in the lipid profiles, the greater the segregation of samples on the respective axis. The resulting scores plot demonstrates a greater separation along principal component 2 for HCVcc-infected samples relative to time-matched mock or UV-HCVcc inoculated controls (Fig. 5), suggesting dramatic changes in the quantitative lipid profile of cells undergoing productive infection. The further segregation of samples along principal component 1 suggests that additional differences in the lipidome occur as infection progresses.

**Figure 6:**
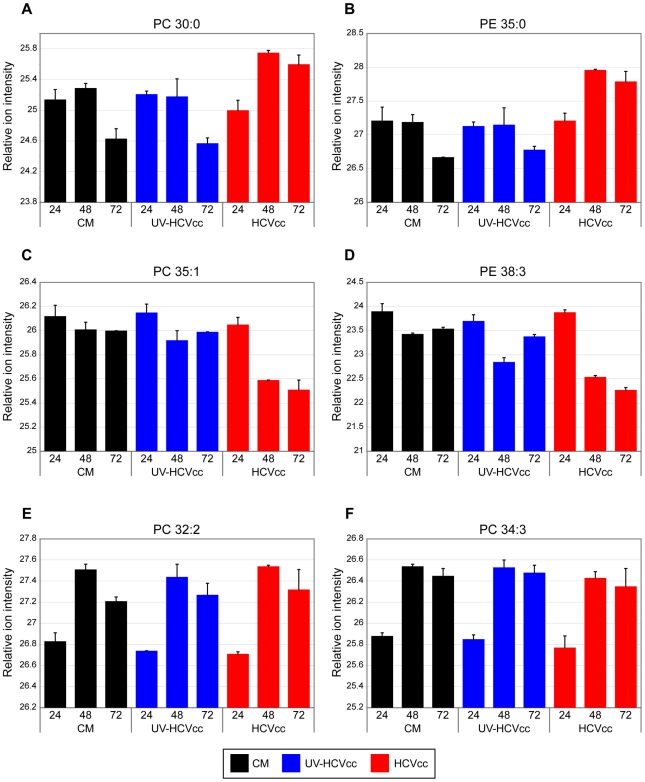
Representative examples of phospholipid species differentially regulated during HCV infection. The relative ion intensity, log 2 scale, and standard deviation is plotted for various phosphatidylcholine (PC) and phosphatidylethanolamine (PE) species monitored 24, 48 and 72 h after infection with either HCVcc (red bars) or UV-HCVcc (blue bars) and using a time-matched mock (conditioned media, CM; black bars) as the reference. Panels A-D (lipid species PC 30∶0, PE 35∶0, PC 35∶1, and PE 38∶3, respectively) provide examples of phospholipid species exhibiting notable changes in abundance during the course of HCV infection. Temporal lipid abundance changes conserved across treatment conditions, panels E and F (lipid species PC 32∶2 and PC 34∶3, respectively), demonstrate the specificity of those perturbations attributed to HCV infection. See Supplementary Table S3 for a complete listing of the lipid features detected in this study.

Consistent with the increased lipogenic activity predicted from our proteome analyses, lipidome analyses revealed a progressive accumulation of several phosphatidylcholine (PC) and phosphatidylethanolamine (PE) species (Supplementary Table S3) that parallels the peak in viral replication observed 72 h post-infection [17]. Representative examples are presented in Figure 6 where the phosphatidylcholine species PC 30∶0 (panel A) and the phosphatidylethanolamine species PE 35∶0 (panel B) are shown to exhibit increased abundances in the HCVcc samples (red bars) at 48 and 72 hours post-infection relative to either the mock (black bars) or UV-inactivated HCVcc (blue bars) samples. These data support a role for lipid species in the viral life cycle, perhaps in the formation of cytosolic lipid droplets and modified membrane compartments for promotion of replication and infectious virus assembly [6]–[13]. Consistent with this, several of the up-regulated proteins identified by the proteomics analysis have been previously detected in purified membrane vesicles containing HCV replication complexes [e.g. N-ethylmaleimide-sensitive factor (NAPG), syntaxin 7 (STX7), annexin A4+A5 (ANXA4+ANXA5), vesicle-associated membrane protein-associated protein B/C (VAPB), peroxisomal enoyl CoA hydratase 1 (ECH1), mitochondrial trifunctional protein (HADHB), NAD(P) dependent steroid dehydrogenase-like (NSDHL)] (Supplementary Table S1) [12].

**Figure 7:**
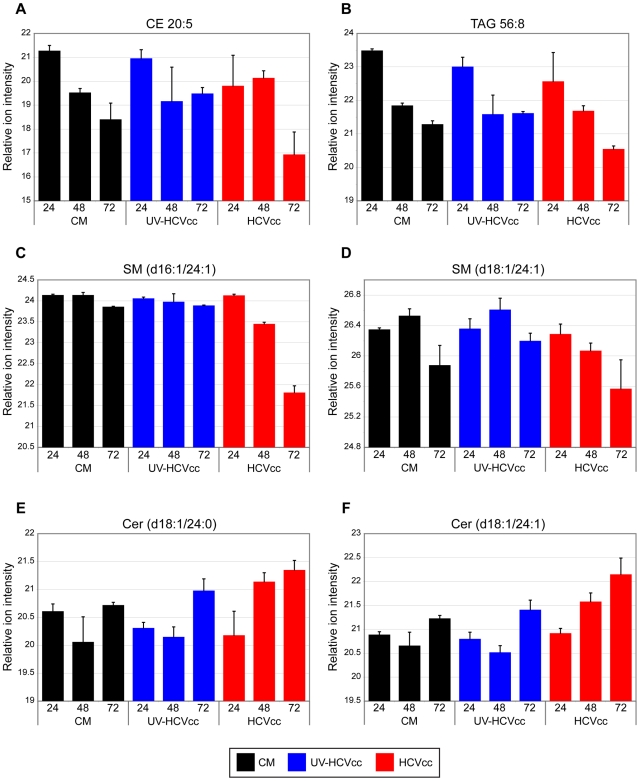
Representative examples of additional major lipid classes differentially regulated during HCV infection. The relative ion intensity, log 2 scale, and standard deviation is plotted for various other lipid classes monitored 24, 48 and 72 h after infection with either HCVcc (red bars) or UV-HCVcc (blue bars) and using a time-matched mock (conditioned media, CM; black bars) as the reference. Panels A-D provide examples of HCV-associated decreases in the relative abundance of cholesterol ester (CE 20∶5), triacylglycerol (TAG 56∶8), and sphingomyelin (SM (d16∶1/24∶1) and SM (d18∶1/24∶1)) species, respectively. This contrasts with the HCV-associated increase in relative abundance of lipotoxic ceramide (CER) species shown in panels E and F (Cer (d18∶1/24∶0) and Cer (d18∶1/24∶1)), respectively. See legend of Figure 5 for details on the experiment representation and Supplementary Table S3 for a complete listing of the lipid features detected in this study.

**Figure 8:**
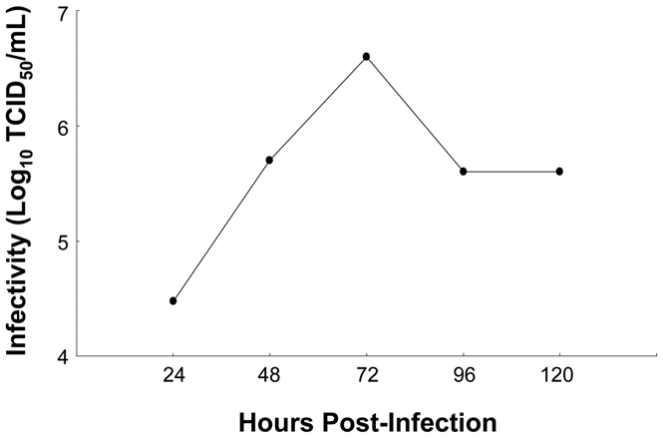
Production of infectious HCV in cell culture. Limiting dilution assays were performed to quantify the amount of infectious HCVcc particles in the cell culture supernatant, measured as 50% tissue culture infectious dose per milliliter (TCID_50_/ml). HCV infectivity accumulated until 72 h, after which a decline occurred that parallels the previously described decrease in HCV RNA levels [17]. The reason for these decreases is unclear, but may be related to a decrease in the number of cells capable of producing high levels of virus.

While some phosphatidylcholine and phosphatidylethanolamine species accumulated during infection, others exhibited a progressive decline in abundance during the course of the HCV life cycle. These included PC 35∶1 and PE 38∶3 whose abundance in HCVcc samples (red bars) decreased over time relative to either mock (black bars) or UV-inactivated HCVcc samples (blue bars) (Fig. 6C and D, respectively). Similar HCVcc-associated temporal abundance decreases were observed within other lipid classes including cholesterol esters (e.g. CE 20∶5, Fig. 7A), triacyglycerols (e.g. TAG 56∶8, Fig. 7B) and sphingomyelins (e.g. SM (d16∶1/24∶1) and SM (d18∶1/24∶1), Fig. 7C and D, respectively). These temporal decreases appear to parallel the accumulation of extracellular infectious virions (Fig. 8), suggesting potential consumption of these lipids in the closely linked processes of very low density lipoprotein (VLDL) and HCV assembly and secretion. Alternatively, the decline in certain sphingomyelin species (e.g. SM (d18∶1/24∶1), Fig.7D) may be explained by their utilization as precursors for the synthesis of reactive ceramides exhibiting concomitant increases in abundance (e.g. Cer (d18∶1/24∶1), Fig. 7F). Ceramides have been shown to play an important role in plasma membrane organization, thereby modulating a variety of cellular processes including apoptosis and endocytosis [44],[45]. Our previously reported global transcriptional profiling of infected Huh-7.5 cells demonstrated that HCVcc may mediate direct cytopathic effects through perturbation of the cell cycle, and that this process may contribute to liver disease progression [17]. The increased abundance of lipotoxic, pro-apoptotic ceramides detected here suggests a previously undescribed link between lipid metabolism and the cytopathic effect [17]. Interestingly, a similar phenomenon has been described in the pathogenesis of Wilson's disease, where copper-induced activation of acid sphingomyelinase results in the accumulation of reactive ceramide species that trigger hepatocyte death and promote fibrogenesis [44]. Alternatively, increased abundance of ceramides may reflect an adaptive host response aimed at minimizing the spread of infection, as ceramides have been reported to have an inhibitory effect on HCV entry [45].

### Novel Protein and Lipid Bottlenecks Identified by Computational Modeling Efforts are Important for HCV-Associated Metabolic Reprogramming

We and others have previously described methods of identifying important genes or proteins based on topological analysis of protein-protein interaction (PPI) and inferred networks [32]–[34]. In this approach, connectivity of proteins or genes in biological networks can provide insight into their relative importance. Briefly, protein or gene “hubs” exhibiting a high degree of connectivity (e.g. connected to many other proteins or genes) and “bottlenecks” exhibiting a high betweeness (e.g. key connectors of sub-networks within a network) represent central points for controlling communication within a network and tend to play an essential role in growth, virulence and targeting by pathogens.

**Figure 9:**
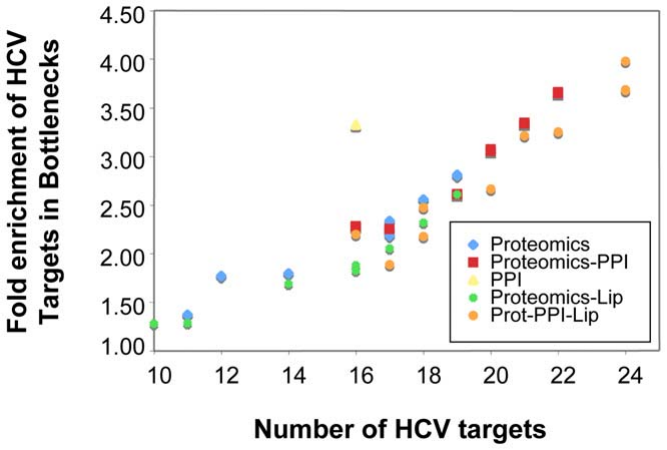
Integration of different network types improves identification of important nodes in HCV infection. Topology of the networks was calculated to identify topological bottlenecks, which were then evaluated for statistical enrichment in proteins known to be targets of HCV proteins by IMAP [46]. The number of identified targets (X axis) is shown against the overall fold enrichment of targets in bottlenecks relative to the rest of the network. The results show that integrating different networks improves the ability to detect these important proteins, and implies that bottlenecks not known to be direct targets of HCV might be playing important roles in the host response process. The networks are: PPI, human protein-protein interactions limited to those between proteins identified by proteomics; Proteomics, correlation-based network from proteomics abundance data; Proteomics-PPI, combined network of proteomics and PPI networks; Proteomics-Lip, combined network of proteomics and lipidomics; Prot-PPI-Lip, combined network of proteomics, PPI and lipidomics.

Here we constructed a protein association network based on correlations between protein abundance such that proteins exhibiting similar abundances (e.g. co-expressed) are connected in the network. We then used the interaction of host proteins with HCV proteins (as determined in IMAP, [46]) as a measure of importance. That is, proteins which are documented targets of direct action by HCV can be considered more important to the process of infection than those with no known viral associations. The statistical enrichment of hub and bottleneck proteins in known HCV targets was determined using a chi-square test. We found that both bottleneck and hub proteins were significantly enriched in HCV targets, showing 2–3 fold enrichment depending on the correlation threshold used for network generation. These computationally revealed proteins were representative of pathways known to be perturbed by HCV, including cholesterol transport, antigen processing and presentation, as well as cell cycle regulation and cell death, further suggesting the validity of this approach. We next integrated the abundance correlation networks with known PPIs, producing a master network of connected cellular processes at multiple levels. This integrated network displays a greater enrichment of HCV targets in bottlenecks and hubs than either of the ‘parent’ networks, and thus identifies more targets with a greater accuracy (Fig. 9). We then integrated the lipidomics profiles into this network using the same correlation approach. Briefly, we calculated the Pearson correlation between the abundance profile of each identified lipid species with a matched profile from each protein. Incorporation of lipids into the integrated network (Fig. 9, circles) improved the number of HCV protein targets identified slightly but, importantly, did not decrease the ability of topological features to identify targets.

**Figure 10:**
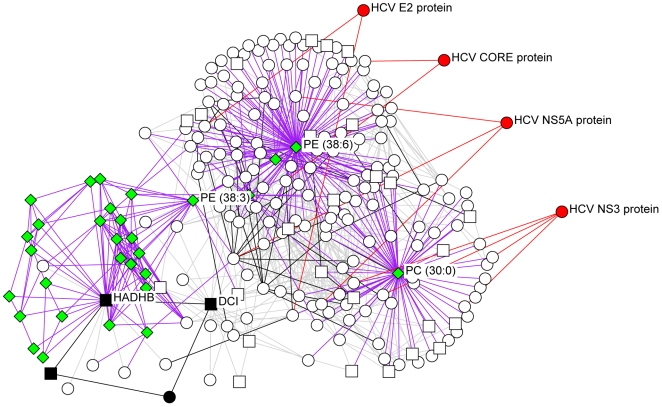
The integrated network surrounding several key bottlenecks identified by computational modeling efforts. The neighbors of bottlenecks in the integrated network (DCI, HADHB, PC 30∶0, PE 38∶3, and PE 38∶6) are shown. Relationships between the proteins and lipid species are grey for proteomics correlation, purple for lipidomics-proteomics correlation, black for protein-protein interactions and red for IMAP relationships. Lipid species are indicated as green diamonds, HCV proteins are red, mitochondrial proteins are squares, and proteins involved in fatty acid β-oxidation are in black.

This analysis provides a number of interesting predictions of important proteins in the host response to HCV infection. Major bottleneck nodes (proteins or lipids) are listed in Supplementary Table S4, and a representative example of an integrated network surrounding several key bottlenecks is shown in Figure 10. Among the bottlenecks are several differentially regulated phospholipids (e.g. PC 30∶0 and PE 38∶3) and two fatty acid oxidation enzymes (HADHB and DCI) (Fig. 10) that were seen to be differentially regulated both in culture and in HCV-infected patients (Fig. 4). Notably, an apparent decline in the relative abundance of these enzymes coincided with the appearance of a cytopathic effect *in vitro* (e.g. 72 h post infection) and the appearance of significant liver injury in patients chronically infected with HCV (e.g. stage 3–4 fibrosis). These findings suggest that HCV-associated targeting of mitochondrial fatty acid oxidation enzymes may contribute to the temporal alterations in cellular metabolic homeostasis that occur during infection and to the impairment of key mitochondrial processes associated with HCV pathogenesis [20].

## Discussion

The increasing availability and application of high-throughput methodologies for gene, protein and metabolite profiling is beginning to provide considerable insight into how viruses modulate intracellular host metabolism in order to meet the needs of the virus growth cycle. For example, a series of elegant studies integrating mRNA expression profiles with intracellular metabolite concentrations demonstrated that human cytomegalovirus (HCMV) institutes its own metabolic program, in part by virus-induced transcriptional changes that modulate the accumulation of select glycolytic and TCA intermediates [47]. Subsequent global kinetic flux measurements revealed a massive up-regulation of nucleotide and fatty acid biosynthetic reactions [48]. Further investigation of this unanticipated increase in fatty acid biosynthesis led to the demonstration that viral replication could be decreased by a pharmacologic inhibitor of acetyl-coenyzme A carboxylase, the enzyme catalyzing the committed step in fatty acid synthesis [48]. Similar metabolic perturbations have been observed in HIV-1-infected cells [49]–[51], where viral-induced reprogramming appears to partition glucose and TCA intermediates toward biosynthetic reactions, supporting, for example, the increased cholesterol production previously predicted by gene expression analyses and confirmed by metabolic labeling studies [52]. These findings demonstrate the importance of complementary transcriptome and proteome studies in investigating virus-induced metabolic changes, and highlight the potential of such studies to elucidate the mechanisms by which viruses usurp cellular metabolic resources and to uncover new avenues/targets for drug discovery.

Here we describe global proteomic and metabolomic profiling studies that provide insight into the metabolic interplay occurring during infection of cultured human hepatoma cells with HCV. Our results indicate that a sequence of protein and lipid abundance changes during the acute phase of HCV infection reflects a disruption of normal metabolic homeostasis, and a shift from energy consuming to energy conserving activities over time. HCV initially reprograms the cell to favor increased glucose fermentation and the partitioning of glycolytic intermediates toward the synthesis of cellular metabolites supporting the viral life cycle. Proteins functioning in oxidative phosphorylation, including components of respiratory complexes I and IV and the proton pump, were also detected. While this finding would seem to contradict a previously reported HCV-mediated impairment of oxidative phosphorylation and accompanying dependence on glucose [53], it is possible that the increased protein abundances observed here reflect a compensatory response. We suspect, however, that the electron transport chain remains at least partially intact, as HCV-infected cells show detectable ATP production from a carbohydrate source requiring oxidative phosphorylation, and limited increases in complex III and IV activity occur even during HCV-associated glucose dependence [53]. Oxidative phosphorylation may elicit lower levels of ATP production not only as a result of impaired mitochondrial respiration *per se*, but also due to the diversion of TCA cycle substrates towards elevated macromolecular biosynthesis during this early phase of infection. Collectively, these findings suggest that HCV-associated glucose dependence may reflect a specific metabolic reprogramming aimed at benefiting both energetic and biosynthetic needs. An analogous situation has been described for cancer cells, where high glycolytic rates occurring in the presence or absence of oxidative metabolism offer the potential for sufficient ATP production while simultaneously providing the cell with intermediates for macromolecular biosynthesis [36],[54].

The trend favoring biosynthesis early in HCV infection was somewhat surprisingly accompanied by an increase in fatty acid oxidation at 24 h. While increased malonyl-CoA, a product of lipogenesis, is expected to impair the uptake of long chain fatty acids via inhibition of carnitine palmitoyl transfersase 1 (CPT1), and thus attenuate fatty acid oxidation, differential regulation of lipogenesis and oxidation can occur through separate pools of malonyl-CoA in the cytosol and mitochondria, respectively [55]. While the relevance of this simultaneous induction in lipid synthesis and catabolism during early HCV infection remains to be determined, we can envision several potential scenarios. Acetyl-CoA produced from fatty acid catabolism may fuel the generation of a new pool of fatty acids populated by constituents essential to the viral life cycle. Alternatively, fatty acid oxidation may be important in maintaining elevated cellular ATP levels supporting energy-dependent biosynthetic processes. Interestingly, a similar phenomenon of increased glucose utilization, *de novo* lipogenesis, and active fatty acid oxidation has been described as part of the thermogenic process in brown adipose and other insulin-sensitive tissues, such as skeletal muscle [56],[57]. Substrate cycling between lipogenesis and lipid oxidation represents a mechanism for protecting muscle tissue against lipotoxicity and glucolipotoxicity [57], and may similarly protect the hepatocyte/liver against the toxic effects of lipid overload, including insulin resistance and lipid-induced cell death. This early induction of fatty acid oxidation may limit the accumulation of ceramide, the appearance of which correlated with HCV-dependent cytopathic effects later in infection [17].

At 48 h post-infection, we observed a decline in the relative abundance of glycolytic enzymes,which apparently reduces macromolecular synthesis and renders the cell dependent on alternative energy sources as infection progresses. This decreased glucose utilization is consistent with the recent report that HCV replication suppresses cellular glucose uptake via down-regulation of cell surface glucose transporters [58]. Host inflammatory responses have been suggested to mediate this effect [58], which is temporally consistent with our previous observation of pro-inflammatory cytokine gene induction [17]. Our failure to detect the resultant protein products presumably reflects their secretion into the cell culture supernatant. The sustained up-regulation of fatty acid and amino acid catabolism appears to account for energy production at this stage of infection, and this switch may channel substrates from synthetic to energetic purposes in the wake of increasing cellular stress. Previous studies have demonstrated that HCV-induced ER stress results in the release of Ca^++^, which can then be transported into mitochondria [53]. This increased recycling of calcium, which coincides with maximum viral protein expression [17],[53], elicits a decrease in mitochondrial membrane potential, elevated reactive nitrogen and oxygen species, and inhibition of respiratory activity [53]. The resulting decline in ATP production necessitates compensatory shifts in an effort to maintain energy homeostasis and cell viability, negatively impacting high-energy processes like lipid, nucleotide and protein synthesis. Consistent with this, our proteomic data demonstrated that the initial increases in translation initiation factors and biosynthetic enzymes was dramatically abated at 48 h. Despite attempts to maintain homeostasis, it is likely that a significant energetic deficit occurs during the elevated viral replication, which, together with oxidative stress, may contribute to the adverse effects of HCV infection on cell proliferation and viability [17].

The protein abundance changes described in this study were typically not accompanied by concomitant gene expression changes, stressing the importance of post-transcriptional regulatory mechanisms during HCV infection [17],[35]. It is interesting to note that, while virus infection is often associated with a global attenuation of host cell translation, HCV-associated impairment of translation initiation factor eIF2α phosphorylation appears to instead increase overall protein synthesis [41],[42],[59]. The molecular basis for selective up-regulation of proteins functioning in metabolic reconfiguration, however, remains to be defined. Interestingly, recent studies have identified an important role for miR-122, the predominant microRNA (miRNA) in liver and Huh-7.5 cells, in regulating lipid metabolism [60],[61]. Antisense-mediated antagonism of miR-122 was shown to decrease fatty acid and cholesterol synthesis, while fatty acid oxidation rates increased, thus favoring a switch to an energy-generating state [61]. Although these changes were attributed to a mild down-regulation (typically less than twofold) of mRNA, the interplay between miRNA abundance and miRNA-mediated mRNA degradation and translational repression is not well understood, and the effects of miR-122 antagonism on translational regulation were not evaluated. It is possible that post-transcriptional mechanisms involving miR-122 contribute to similar alterations in metabolic phenotype during HCV infection. In this regard, miR-122 is known to bind the 5′ UTR of HCV RNA and enhance replication [62]. Increasing intracellular HCV RNA concentrations may therefore result in sequestration and progressive depletion of miR-122, thereby interfering with lipogenesis and contributing to the reprogramming of hepatocellular metabolism and bioenergetics. Although our steady-state measurements do not allow us to distinguish between perturbations in protein stability and protein synthesis, the recent development of new proteomic approaches, such as pulsed stable isotope labeling with amino acids in cell culture; pSILAC, offers opportunities to evaluate the contribution of translational regulation to HCV-associated changes in the host proteome [63].

Differential regulation of a variety of lipid species was predicted by our proteome studies and subsequently confirmed by lipidomic analyses. The increased abundance of select phospholipids may reflect their role as important constituents in the various structural entities supporting viral replication, including the lipid droplet and membranous replicase compartments. In contrast, the decline in other phospholipid species and lipid classes (e.g. sphingomyelin, triacylglycerol) may reflect their incorporation into lipoprotein associated viral particles and potentially important roles in infectivity. Although HCV-associated cholesterol and sphingolipid have been shown to play a critical role in infection [16], the precise virion lipid composition, including molecular species of each class and relationship to virion maturation, is unknown. Recent mass spectrometry based analyses of the HIV lipidome have identified a broad repertoire of lipids within the virion [16],[64], as well as highlighted a key role for raft-like microdomains in virion budding and identified select phosphatidylserine species, which may represent viable therapeutic targets for modulating membrane composition and infectivity [64],[65]. Our studies of HCV-infected cells provide a valuable reference for use dissecting the role of various host cell lipid species in the viral life cycle.

In summary, the results of our integrative genomics efforts provide new clues for understanding the role of metabolic reprogramming during the acute phase of HCV infection. This study is unique in that it provides the first demonstration of profound modifications in the proteome indicating that a temporal sequence of metabolic alterations occurs during HCV infection, and describes a previously un-identified key role for post-transcriptional regulatory mechanisms in this metabolic re-routing. Significantly, the presence of metabolic perturbations was confirmed by measurement of lipid metabolite levels. Concomitant increases in intracellular lipids and viral loads were observed to occur subsequent to the early induction of multiple lipogenic proteins, including enzymes [e.g. acetyl coa carboxylase (ACAA) and fatty acid synthase (FASN)] whose pharmacologic inhibition was previously shown to hinder HCV replication in a stable HCV full-length replicon system [66]. Taken together, we believe that these observations provide “proof of principle” for the utility of integrative functional genomics studies to shed new light on processes of functional significance during HCV infection and pathogenesis.

Typical of high-throughput functional genomics studies, our global proteome and lipidome analyses have led to many new and interesting hypotheses that warrant further investigation. A major challenge in translational hepatology research is to integrate the vast amounts of data collected from these high-throughput approaches into a single systems-level view that allows identification and prioritization of potential targets for novel antiviral therapeutics. To address this challenge we have used computational modeling approaches to begin constructing a molecular interaction network that describes key components and interactions associated with HCV infection. These efforts have led to several novel observations, including the identification of two mitochondrial fatty acid oxidation enzymes, trifunctional enzyme β subunit (HADHB) and 3,2-trans-enoyl-CoA isomerase (DCI), as network bottlenecks predicted to play a key regulatory role in HCV-associated metabolic reprogramming. Interestingly, the modulation of fatty acid oxidation has been linked to the suppression of virus replication and reduction of cytopathic effects in an *in vitro* model of persistent measles virus infection, and a potential role in evasion of the immune response has been suggested [67]. To further investigate the role of HADHB and DCI in the HCV life cycle we are now employing standard RNA interference (RNAi) methods to generate stable shRNA knockdown clones in retinoic acid-induced gene 1 (RIG-I) competent Huh7 cells as well as Huh-7.5 cells in order to evaluate the impact on virus replication, assembly and secretion. Observation of a phenotypic effect would open the door to several new and exciting avenues of investigation, including further exploration of a potential mechanism involving interactions between viral replication, fatty acid oxidation and host innate immune responses in the establishment of chronic HCV infection. The results of such studies will also facilitate efforts to link clinical functional genomics profiles with pathway-specific signaling and gene/protein/metabolite expression and function. Finally, these phenotypic perturbations would provide a starting point for exacting the systems biology approach, which involves iterative rounds of experimental manipulation and high-throughput profiling for refinement of model predictions and a more insightful multi-dimensional view of virus-host interactions [68]. Interpretation of this refined global view of HCV-mediated cellular reprogramming will provide valuable guidance in the design of novel antiviral therapeutics aimed at modifying HCV infection and/or pathogenesis.

## Supporting Information

Table S1 Comprehensive list of the 2,418 proteins quantified by proteomic analysis of Huh-7.5 cells infected with HCV. Abundance ratios were monitored 24, 48 and 72 h after infection with either HCVcc (chimeric HCV 2a virus, J6/JFH-1) or UV-HCVcc (UV-inactivated chimeric HCV 2a virus, J6/JFH-1) and using a time-matched mock (conditioned media) as the reference. Final protein abundance ratios and accompanying P-values were calculated in Elucidator by combining data from the 5 strong cation exchange (SCX) fractions generated and analyzed for each sample. Also reported here are the log protein abundance ratio, fold change, log error, and XDev (log ratio/log error). A total of 495 proteins were differentially regulated (>1.5-fold change, P-Value<0.05) in at least two experiments and are highlighted in bold. These proteins are arranged in order of the fold-change observed in the HCVcc sample at 24 h post-infection.
(1.71 MB XLS)

Table S2 Select list of altered protein abundances reflecting perturbations in host metabolism and adaptive responses during HCV infection. Fold-change in protein abundance and accompanying P-values were calculated in Elucidator as described in Table S1. Differentially regulated proteins (>1.5-fold change, P-Value<0.05) exhibiting statistically significant increases in abundance are highlighted in red and those exhibiting statistically significant decreases in abundance are highlighted in green. Functional categories represented include glycolysis, the pentose phosphate pathway, and the tricarboxylic acid (TCA) cycle whose constituents are listed in their order of appearance in the appropriate pathway. For all other functional categories typically representing a broader spectrum of activities including pyruvate metabolism, oxidative phosphorylation, glutamate metabolism, fatty acid oxidation, nucleotide biosynthesis and homeostasis, lipogenesis, chaperones, and NRF2 stress response the proteins have been arranged in order of the fold-change observed in the HCVcc sample at 24 h post-infection.
(0.07 MB XLS)

Table S3 Select list of 272 lipid species exhibiting significant differences across conditions and time points (ANOVA P<0.05). Mass to charge ratio (M/Z), normalized elution time (NET), log 2 average abundances (mean) and standard deviations (sd) are presented for each condition (CM: conditioned media, UV-HCVcc: UV-inactivated chimeric HCV 2a, HCVcc: chimeric HCV 2a virus) and time point. Also reported are the overall P-values from ANOVA analysis performed on minimum observation data (a feature was required to be observed in at least two out of three conditions (CM, UV-HCVcc, HCVcc) and there must be duplicate measurements for the two out of three conditions). P-values listed as 0.000 are equivalent to p<0.001. NA indicates where missing values exist. Among the 272 features exhibiting statistically significant differences between treatment conditions and/or time points, 73 lipid species were identified by matching to a lipid AMT tag database or fragmentation information collected via targeted MS/MS analyses. Identity abbreviations were made for phoshatidylcholine (PC; O- fatty acid chain number means that an alkyl acyl linkage to the glycerol chain is present for the respective PC), sphingomyelin (SM), ceramide (Cer), triacylglycerol (TAG), and cholesterol ester (CE). The notation further indicates total number of carbons and double bonds however it does not discern redundancy associated with varying fatty acid composition for the same molecular weight.
(0.07 MB XLS)

Table S4 Top 5% of the bottlenecks identified from the combined lipidomics, proteomics inferred and protein-protein interaction network. Accompanying notes about the known or implicated roles of the identified bottlenecks in HCV response is also included.
(0.03 MB XLS)
